# Ethnic spice use and perceived health benefits among women in a Suburb of the Greater Accra Region of Ghana

**DOI:** 10.4314/ahs.v22i4.28

**Published:** 2022-12

**Authors:** Matilda Asante, Precious Asante, Portia Nkumsah-Riverson, Anna Amokao-Mensah, Yauniuck Dogbe

**Affiliations:** University of Ghana College of Health Sciences, Department of Dietetics, School of Biomedical and Allied Health Sciences

**Keywords:** Spices, health benefits, perception, flavour

## Abstract

**Background:**

Spices have a long history of both culinary use and health benefits. Ethnic spices are spices specific to a particular geographic location. In Ghana, there are varying perceptions and uses of ethnic spices which are mostly not documented.

**Objective:**

To assess the use and perceived health benefits of ethnic spices among women in Accra, Ghana.

**Methods:**

A cross-sectional study was conducted among 370 women in randomly selected churches in Accra, Ghana. A semi-structured questionnaire was used to obtain sociodemographic data and information on ethnic spice use. Chi-square test was used to determine the association between socio-demographic characteristics of participants and perceived health benefits of ethnic spice usage.

**Results:**

The most commonly consumed spices out of the 19 spices explored in this study were onion (98.4%), garlic (97.6%), ginger (96.8%) and chili pepper (84.1%). Majority of the participants perceived ginger (74.6%) and garlic (73.8%) as having health benefits. Age (p=.001) and self-reported health status (p=0.010) were significantly associated with perceived health benefits of the spices.

**Conclusion:**

Onion, garlic, ginger and chili pepper were the four most commonly consumed ethnic spices. Age and self-reported health status of participants influenced perceived health benefits of ethnic spice usage.

## Introduction

Spices are ingredients that are commonly used to improve aroma and visual appeal of foods, enhance taste and also act as preserving agents.^1^ In terms of composition, a spice may comprise any part of a plant e.g. the bark, buds, fruits, leaves, roots, seeds, flower or the entire plant.^2^ Spices have many other benefits aside from their long history of use for culinary purposes.^1^, ^3^ A strong relationship has been established between usage of certain ethnic spices and decreasing health risks.^4^, ^5^. Some of the health benefits of spices include lowering cholesterol levels, having modest reduction in blood pressure and blood glucose, relieving arthritic knee pain, aiding in digestion, improving respiratory health and anti-microbial activity.^4^, ^6^ The use of spices is not a modern-day phenomenon but a practice that is as old as the history of mankind. Currently, there is an increase in the use of spices because of exposure to different dishes from other countries through travel and migration across national boundaries.^7^ Furthermore, the global herb and spice industry, valued at approximately 4 billion US dollars, continues to grow,^8^ reflecting a high usage of spices, worldwide.^9^ There is also greater dissemination of nutrition information through the mass media, and the sudden insistence of the consuming public for more spicy, tropical, healthy and convenient foods.^7^ The interest in the benefits of spices is due to chemical compounds such as phenylpropanoids, terpenes, flavonoids and anthocyanin found in them.^10^ All these compounds confer different properties to the spices such as antimicrobial and antioxidant activity.^10^

Ghana is greatly endowed with many spices with the potential of being used in developing good seasoning.^11^ Some natural spices in Ghana are fermented African locust beans, bay leaf, calabash nutmeg, cloves, African black pepper, anise seeds, rosemary and many more.^12^ In Ghana, the use of spices by households is a common phenomenon, with anecdotal evidence suggesting that these spices are used because of their potential health benefits. Information on ethnic spice is passed on by word of mouth over several generations in Ghana.^7^ Further, there is limited documented information on spices and their use (women who cooked with ethnic spices or applied a named spice for other purposes other than in foods), and perceived health benefits by women who are the ‘gatekeepers’ at home. Documentation of such information would help nutrition care professionals in counselling patients on the appropriate use of spices for culinary purposes as well as complementary alternative medicine, thus the need for this study.

## Methods

### Study design and site

This was a cross-sectional study conducted at selected churches in Korle Gonno, an electoral area in the Ablekuma-South Sub Metropolis of the Greater-Accra Region of Ghana.^13^ Korle Gonno is one of Accra's coastal communities, located to the west of Korle Lagoon and the densely populated districts of James Town and Usher Town. The area has a total population size of 27,826 comprising of 13,400 females.^14^ Most (63%) of the households in Korle Gonno are headed by females and about ninety-five per cent of the people living in Korle Gono are Christians.15 Churches in the area consist of 13 Pentecostal/Charismatic churches and four Mainline churches (Anglican, Presbyterian, Methodist, and the Seventh Day Adventist church).

### Study population, sample size and sampling technique

Participants of the study were women from selected churches in Korle Gonno, Accra. Three churches were randomly selected from the seventeen churches from the locality.

The total enumeration technique was used to recruit all eligible women from the various churches. Participants who refused to participate were excluded. A minimum sample size of 384 was calculated using Cochran16 formula at a 95% confidence interval and margin of error of 5%.

### Procedure for data collection

A semi-structured questionnaire was adopted from a similar study in the US and a part of India, 9, 17 and modified to suit the study population. The questionnaire was pre-tested among 40 women in a similar population. The questionnaire was used to obtain the demographic (age, occupation, ethnicity, and educational level) and socioeconomic (monthly income) information of participants. The questionnaire also explored the commonly consumed ethnic spices, reasons for use and perceived health benefits among others.

### Data Analysis

Data collected was entered and analysed using the Statistical Package for Social Sciences (SPSS) version 23.0 software. Descriptive statistics (frequencies and percentages) were used to summarize categorical variables. Association between sociodemographic characteristics and perceived health benefits of ethnic spices was examined with the chi-square test. Level of significance was set at p<0.05.

### Ethical issues

The study was approved by the Ethics and Protocol Review Committee of the School of Biomedical and Allied Health Sciences, College of Health Sciences, University of Ghana (SBAHS-DT/10564489/SA/2018-2019). Written informed consent was obtained from all the participants prior to data collection.

## Results

A total of 370 women participated in the study (96.4% response rate). Details of the sociodemographic characteristics of the study participants are presented in Table 1. About a third of the participants (30.8%) in this study were within the 18 -28 years old age bracket. About a third of the participants reported that they had very good health status with less than three percent reporting otherwise. Nearly half of them (49.1%) had attained tertiary level education. Less than half of the participants (45.7%) earned less than GHC500.00 monthly ($88), while 12.4% earned above GHC1,000 to GHC1,500 $175 – $263) (Table 1).

**Table 1:** Socio-demographic characteristic of participants (N=370)

Variables	Frequency (n)	Percentage (%)
**Age Group (Years)**		
18–28	114	30.8
29–39	59	15.9
40–49	62	16.8
50–59	59	15.9
60 and above	76	20.6
**Reported Health Status**		
Excellent	95	25.7
Very good	111	30.0
Good	84	22.7
Fair	70	18.9
Poor	10	2.7
**Educational status**		
Primary	31	8.4
Junior High School	80	21.6
Senior High School	77	20.8
Tertiary	182	49.1
**Income level (Ghana Cedis)**		
<500	169	45.7
500–1000	84	22.7
>1000–1500	46	12.4
>1500	71	19.2
**Occupation**		
Artisans/tradesperson/unskilled worker	186	50.3
Nurse/doctor/dietitian/dentist/Accountant	69	18.6
Clerical/Audit/Accounts/Office worker	29	7.8
Student	57	15.4
Retired	27	7.3
Unemployed	2	0.5

USD/Ghana cedi exchange rate: 1 US Dollar = 5.7000 Ghanaian cedi as of 12/31/2019

### Types of spices and use

**Figure 1:**
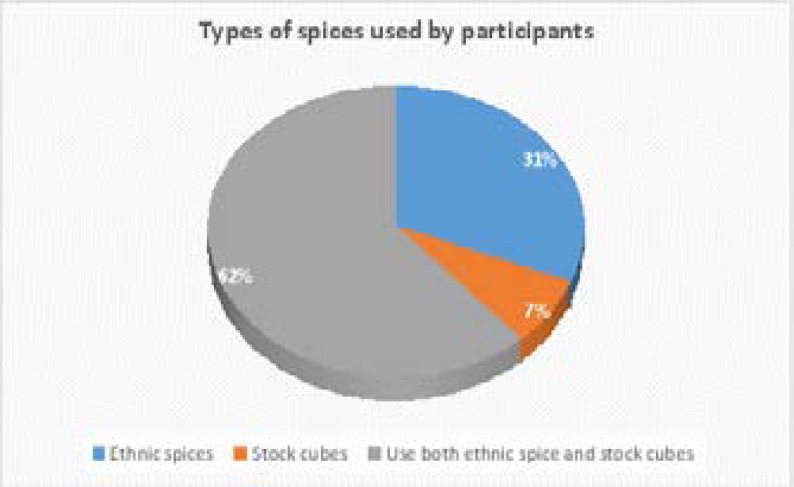
Types of spices used by participants

**Figure 2:**
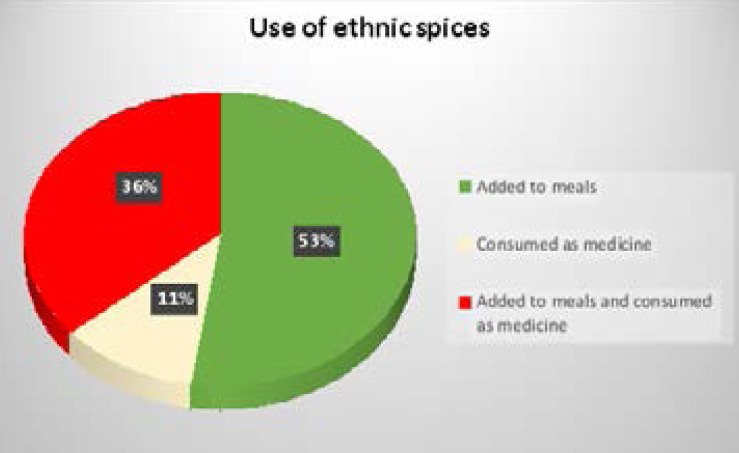
How participants used ethnic spices

A little over sixty percent of the women used ethnic spices together with stock/bouillon cubes while 31.0% used only ethnic spices (figure 1). More than half of the participants (52.0%) reported adding ethnic spices to their meals (figure 2).

### Sources of information about ethnic spices

Information on spices was obtained from several sources, with close to 70% from non-health professionals. More than 80% knew that using ethnic spice offered health benefits, and 48.6% of them were eager to know more about the health benefits of ethnic spices. Less than a third of the participants (27.8%) believed that ethnic spices were free from adulterants (Table 2).

**Table 2:** Knowledge or information about ethnic spices (N=370)

Variables	Frequency (n)	Percentage (%)
**Ever encouraged by a health professional to** **consume ethnic spices**		
Yes	117	31.6
No	253	68.4
**Source of information on ethnic spices**		
Registered dietician	52	14.1
Family	255	68.9
Friends	32	8.6
Social media	7	1.9
Magazines	12	3.2
Other health professionals	5	1.4
Other (internet, school, radio & TV)	7	1.9
**Ethnic spices provide some health benefits**		
Yes	313	84.6
No	20	5.4
Do not Know	37	10.0
**Interested in learning about the health benefits of** **ethnic spices**		
Very interested	180	48.6
Interested	96	25.9
Neutral	63	17.0
Disinterested	16	4.3
Very disinterested	15	4.1
**Is excessive use of ethnic spices bad for health?**		
Yes	185	50.0
No	106	28.6
Do not know	79	21.4
**Belief that ethnic spices are free from adulterants**		
Yes	103	27.8
No	118	31.9
Do not know	149	40.3

### Commonly consumed ethnic spices and reasons for use

The fourteen (14) commonly consumed ethnic spices out of the 19 spices explored in this study are shown in table 3. Onion, garlic, ginger and chili pepper were the four most commonly consumed spices with the least consumed being Aridan and black pepper.

**Table 3:** Commonly consumed ethnic spices by respondents

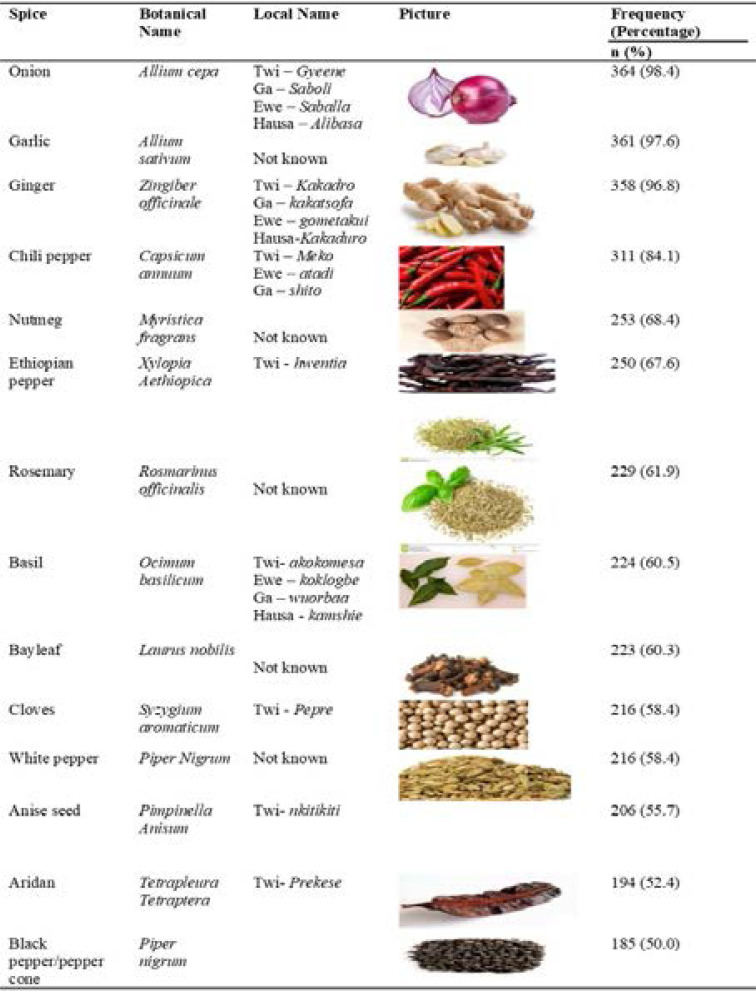

Participants reported that they mainly used onion because of its taste (68.1%), flavour (58.6%), nutritious quality (48.1%) and its medicinal properties (41.6%). Garlic was also used mainly for its flavour (60.0%), medicinal properties (59.7%), taste (55.7%) and nutritious quality (54.9%). Furthermore, ginger was used by most of the participants because of its flavour (64.1%), taste (58.4%), medicinal properties (55.4%) and nutritious quality (53.2%), while chili pepper was used mostly for its taste (51.4%). Black pepper, the spice which was least used, was used mainly because of its taste (22.2%) and flavour (21.6%) (Table 4).

**Table 4:** Reasons for ethnic spice use

Spice	Medicinal n (%)	Nutritious n (%)	Flavour n (%)	Taste n (%)	Texture n (%)	Colour n (%)
Onion	154 (41.6)	178 (48.1)	217 (58.6)	252 (68.1)	16 (4.3)	11 (3.0)
Garlic	221 (59.7)	203 (54.9)	222 (60.0)	206 (55.7)	9 (2.4)	7 (1.9)
Ginger	205 (55.4)	197 (53.2)	237 (64.1)	216 (58.4)	19 (5.1)	12 (3.2)
Chili pepper	42 (12.2)	80 (21.6)	114 (30.8)	190 (51.4)	5 (1.4)	18 (4.9)
Nutmeg	63 (17.0)	79 (21.4)	208 (56.2)	106 (28.6)	9 (2.4)	3 (0.8)
Ethiopian pepper	175 (47.3)	101 (27.3)	139 (37.6)	100 (27.0)	12 (3.2)	12 (3.2)
Rosemary	56 (15.1)	83 (22.4)	178 (48.1)	115 (31.1)	7 (1.9)	2 (0.5)
Basil	68 (18.4)	79 (21.4)	159 (43.0)	106 (28.6)	11 (3.0)	3 (0.8)
Bay leaf	66 (17.8)	85 (23.0)	146 (39.5)	94 (25.4)	7 (1.9)	12 (3.2)
Cloves	105 (28.4)	92 (24.9)	125 (33.8)	104 (28.1)	6 (1.6)	9 (2.4)
White pepper	37 (10.0)	58 (15.7)	156 (42.2)	112 (30.3)	6 (1.6)	6 (1.6)
Anise seed	77 (20.8)	85 (23.0)	132 (35.7)	88 (23.8)	9 (2.4)	1 (0.3)
Aridan	122 (33.0)	97 (26.2)	92 (24.9)	68 (18.4)	4 (1.1)	4 (1.1)
Black pepper	36 (9.7)	54 (14.6)	80 (21.6)	82 (22.2)	4 (1.1)	6 (1.6)

### Perceived health benefits of ethnic spices

Majority of participants perceived that ginger (74.6%), garlic (73.8%), onion (63.5%) and Ethiopian pepper (64.1%) had some health benefits (Table 5).

**Table 5:** Do you think any of these spices have health benefits (N=370)

Spices	Yes		No		Do not know	
	n	(%)	n	(%)	n	(%)
Onion	235	(63.5)	15	(4.1)	120	(32.4)
Garlic	273	(73.8)	13	(3.5)	84	(22.7)
Ginger	276	(74.6)	17	(4.6)	77	(20.8)
Chili pepper	141	(38.1)	31	(8.4)	198	(53.5)
Nutmeg	143	(38.6)	28	(7.6)	199	(53.8)
Ethiopian pepper	237	(64.1)	20	(5.4)	113	(30.5)
Rosemary	137	(37.0)	33	(8.9)	200	(54.1)
Basil	141	(38.1)	18	(4.9)	211	(57.0)
Bay leaf	136	(36.8)	21	(5.7)	213	(57.6)
Cloves	142	(38.4)	22	(5.9)	206	(55.7)
White pepper	113	(30.5)	41	(11.1)	216	(58.4)
Anise seed	143	(38.6)	19	(5.1)	208	(56.2)
Aridan	155	(41.9)	16	(4.3)	199	(53.8)
Black pepper	107	(28.9)	17	(4.6)	246	(66.5)

### Relationship between socio-demographic characteristics and health benefits

Age (p=0.001) and self-reported health status (p=0.010) of participants were significantly associated with health benefits of spice use (Table 6). Of the group that perceived those ethnic spices had health benefits, about a third (31.3%) were between the ages of 18 and 28 years. Furthermore, the highest proportion of participants who were of the view that ethnic spices had health benefits were those who reported very good (31.0%) or excellent (26.2%) health status. Educational status and income level of the women were not associated with perceived health benefits of spice use.

**Table 6:** Association between socio-demographic characteristics and health benefits

Variables	Do ethnic spices provide some health benefits			X^2^	P-value
	Yes	No	Do not know		
	n (%)	n (%)	n (%)		
**Age groupings** **(years)**					
18–28	98 (31.3)	6 (30.0)	10 (27.0)		
29–39	57 (18.2)	2 (10.0)	0 (0.0)	30.89	0.001*
40–49	47 (15.0)	3 (15.0)	12 (32.4)		
50–59	48 (15.3)	6 (30.0)	5 (13.5)		
60 and above	63 (20.1)	3 (15.0)	10 (27.0)		
**Educational status**					
Primary	5 (4.3)	17 (11.6)	9 (8.3)		
Junior High School	23 (20.0)	20 (13.7)	37 (33.9)		
Senior High School	28 (24.3)	28 (19.2)	21 (19.3)	5.69	0.337
Tertiary	59 (51.3)	81 (55.5)	42 (38.5)		
**Income level** **(Ghana cedi)**					
<500	47 (40.9)	64 (45.7)	58 (50.4)		
500–1000	25 (21.7)	45 (32.1)	12 (10.4)		
>1000–1500	16 (13.9)	13 (9.3)	19 (16.5)	2.81	0.423
>1500	27 (23.5)	18 (12.9)	26 (22.6)		
**Self-reported** **health status**					
Excellent	82 (26.2)	4 (20.0)	9 (24.3)		
Very good	97 (31.0)	10 (50.0)	4 (10.8)		
Good	69 (22.0)	0 (0.0)	15 (40.5)	19.97	0.010*
Fair	56 (17.9)	5 (25.0)	9 (24.3)		
Poor	9 (2.9)	1 (5.0)	0 (0.0)		

Chi square test

* significant at p<0.05

## Discussion

In recent years, there has been growing interest in the use of ethnic spices for medicinal and therapeutic purposes, especially to treat various chronic conditions, reduce disease symptoms, and aid in treatment and management of common health problems. Consumers' usage, perceptions and knowledge about spices vary in different populations. Therefore, the purpose of this study was to assess ethnic spice usage and perceived health benefits among women in a town in the Greater Accra region, Ghana.

Majority of the participants were within the young age bracket (18 -28 years), which confirms a previous report that this area was dominated by a younger generation.^15^ More than half of the participants in the study reported that they had very good or excellent health status. This is probably because most of the participants were the young people. This finding is also in agreement with a WHO report which stated that the youth in Africa were healthy.^18^

More than half of the participants stated that they obtained information about spices from family members. This confirms a previous report by Gadegbeku et al., 7 that information on ethnic spice is passed on by word of mouth over several generations in Ghana. It also shows the important role of culture in influencing food habits. A few of the participants (1.4%) reported that their source of information was from other health professionals besides dieticians. This highlights the need for documentation of such information to help nutrition care professionals in counselling patient on the appropriate use of spices for culinary purposes as well as evidence-based complementary alternative medicine. Nearly half of the participants in this study stated that they were very interested in learning about ethnic spices. Dietary education by health professionals should also emphasize the benefits of using ethnic spices for culinary and health purposes. The recent increase of television and radio advertisements on the use of chemical-based artificial spices as ‘great’ flavour and taste enhancers may be detrimental to the health of consumers.

Majority of the commonly consumed ethnic spices were used because of their flavour and taste. Consistent with findings from Gadegbeku and colleagues^7^ among food vendors in Ghana, the most commonly consumed spices noted in this study were onions, garlic, ginger, and chili. Onion emerged as the most common spice that was used by the participants because of its taste and flavour. Pareek, Sagar, Sharma and Kumar^19^ noted similar reasons (i.e., taste, aroma and flavour) for the use of onions. Some participants in our study (41.6%) used onions for medicinal purposes. Onion and its essential extracts have been used in some clinical studies to lower serum cholesterol levels and reduce fibrinolytic activities.^20^, ^21^ Garlic is a well-known and valuable spice for culinary purposes.^22^, ^23^ For instance, it was reported among the commonly consumed spices in the United States, India and Bangladesh.^9^, ^17^ In this present study, garlic was the second most commonly consumed spice. Participants reported that they used it mainly because of its flavour, medicinal properties, taste and nutritional quality. Other studies have also reported utilization of garlic for its flavour and taste, 22 medicinal properties, 9, 24, 25 and nutritional value.^26^ In terms of its medicinal properties, research shows that garlic acts as an essential resource in immune boosting, wound healing, improvement of blood circulation and blood thinning.^22^, ^24^, ^27^ Ginger was recognized by the participants as a valuable spice for flavour, taste, medicinal and nutritional value. Scientific literature provides evidence that ginger has a number of health-promoting properties, comprising of anti-emetic, antiviral, antibacterial, anticoagulant, and anti-inflammatory among others.^28^, ^29^, ^30^ Although many studies have focused on the medicinal uses and benefits of ginger, it is also recognized globally as a flavouring agent.^28^

Some studies have reported numerous uses of chili pepper as a spice, vegetable, flavour enhancer, colouring agent, aroma, and component in herbal medicines formulations. 31, 32 In this study, chili pepper was used by participants mainly for taste and not for flavour or its medicinal properties. This could probably be due to the heat sensation chili pepper evokes in the mouth when consumed. 31 Ghana, like many other West African countries has a cuisine that highlights spicy stews and condiments. This spice is therefore used as a base in many Ghanaian dishes. Chili is also reported to be the most commonly used spice in Sri Lanka and Bangladesh.^33^ An Ethiopian study also showed that the major seed spices used by people of Bahir Dar City were garlic, fenugreek, basil, thyme and turmeric.^23^

Nutmeg was commonly used by the participants because of its flavour. This could probably be the reason why nutmeg is used in the preparation of many foods.34 Participants also used rosemary, basil, bay leaf, cloves, white pepper, anise seed, and black pepper mostly because of their flavour. Similar reasons for use of these spices were observed in other studies.^35^, ^36^, ^37^

The reason behind the use of Ethiopian pepper by the participants was for its medicinal properties. Ethiopian pepper is used medicinally as a cough medicine, pain killer and as a purgative.^38^, ^39^. In Africa, it is used to treat bronchitis, dysentery, stomach ache, wounds, boils, and other ailments in traditional medicine.^40^ Ethiopian pepper also has antioxidant, 38, 41 hypolipidemic and hypoglycaemic properties.^41^ Evidence of other health benefits of Ethiopian pepper including anti-inflammatory, antimicrobial, anti-arthritic among other medicinal properties is also well documented.^22^, ^39^, ^40^, ^42^ Participants also indicated the use of Aridan for medicinal purposes. Aridan has been reported by many studies to have medicinal effects like wound healing, antimicrobial effect, hypoglycaemic, ant inflammatory, hypotensive, antiulcerative, molluscidial and many others.^43^, ^44^, ^45^

About a third of the women believed that ethnic spices were free from adulterants. Similarly, a study done in a district in India, which assessed consumer's perception on organic spices found that these spices were perceived as free from toxins, chemicals, and pesticides.^17^ In addition, the participants indicated that organic spices were healthy and reduced the risk of chronic diseases rather than conventionally grown ones.^17^

A study done in a part of India, showed that monthly household income and educational levels affected the use of spice, quality of the spices and their cost persuasion. 17 However, in the same study, participants' perception of spices in relation to its health properties were not influenced by age, educational level, occupation and household income in the India study.^17^ In contrast, age (p=0.001), and health status (p=0.010) of participants were significantly associated with perceived health benefits in this study.

## Limitation

Findings of this study were based on participants' self-report. This might have introduced some form of recall bias.

## Conclusion

Onion, garlic, ginger and chili pepper were the four most commonly consumed ethnic spices in this study. The main reason for use of most of the spices was to impart flavour. Majority of the participants perceived ginger and garlic as having health benefits, and as such, were efficacious in preventing diseases and promoting health. Age and self-reported health status of participants were significantly associated with perceived health benefits of ethnic spices. Nutrition care professionals should educate clients/families on the benefits of using ethnic spices for culinary purposes. Furthermore, awareness on ethnic spice use should be increased nationwide using the mass media.
